# Deep Learning–Based Electrocardiogram Model (EIANet) to Predict Emergency Department Cardiac Arrest: Development and External Validation Study

**DOI:** 10.2196/67576

**Published:** 2025-02-28

**Authors:** Shao-Chi Lu, Guang-Yuan Chen, An-Sheng Liu, Jen-Tang Sun, Jun-Wan Gao, Chien-Hua Huang, Chu-Lin Tsai, Li-Chen Fu

**Affiliations:** 1 Department of Computer Science and Information Engineering National Taiwan University Taipei Taiwan; 2 Department of Emergency Medicine Far Eastern Memorial Hospital Taipei Taiwan; 3 Department of Emergency Medicine National Taiwan University Hospital and National Taiwan University College of Medicine Taipei Taiwan

**Keywords:** cardiac arrest, emergency department, deep learning, computer vision, electrocardiogram

## Abstract

**Background:**

In-hospital cardiac arrest (IHCA) is a severe and sudden medical emergency that is characterized by the abrupt cessation of circulatory function, leading to death or irreversible organ damage if not addressed immediately. Emergency department (ED)–based IHCA (EDCA) accounts for 10% to 20% of all IHCA cases. Early detection of EDCA is crucial, yet identifying subtle signs of cardiac deterioration is challenging. Traditional EDCA prediction methods primarily rely on structured vital signs or electrocardiogram (ECG) signals, which require additional preprocessing or specialized devices. This study introduces a novel approach using image-based 12-lead ECG data obtained at ED triage, leveraging the inherent richness of visual ECG patterns to enhance prediction and integration into clinical workflows.

**Objective:**

This study aims to address the challenge of early detection of EDCA by developing an innovative deep learning model, the ECG-Image-Aware Network (EIANet), which uses 12-lead ECG images for early prediction of EDCA. By focusing on readily available triage ECG images, this research seeks to create a practical and accessible solution that seamlessly integrates into real-world ED workflows.

**Methods:**

For adult patients with EDCA (cases), 12-lead ECG images at ED triage were obtained from 2 independent data sets: National Taiwan University Hospital (NTUH) and Far Eastern Memorial Hospital (FEMH). Control ECGs were randomly selected from adult ED patients without cardiac arrest during the same study period. In EIANet, ECG images were first converted to binary form, followed by noise reduction, connected component analysis, and morphological opening. A spatial attention module was incorporated into the ResNet50 architecture to enhance feature extraction, and a custom binary recall loss (BRLoss) was used to balance precision and recall, addressing slight data set imbalance. The model was developed and internally validated on the NTUH-ECG data set and was externally validated on an independent FEMH-ECG data set. The model performance was evaluated using the *F*_1_-score, area under the receiver operating characteristic curve (AUROC), and area under the precision-recall curve (AUPRC).

**Results:**

There were 571 case ECGs and 826 control ECGs in the NTUH data set and 378 case ECGs and 713 control ECGs in the FEMH data set. The novel EIANet model achieved an *F*_1_-score of 0.805, AUROC of 0.896, and AUPRC of 0.842 on the NTUH-ECG data set with a 40% positive sample ratio. It achieved an *F*_1_-score of 0.650, AUROC of 0.803, and AUPRC of 0.678 on the FEMH-ECG data set with a 34.6% positive sample ratio. The feature map showed that the region of interest in the ECG was the ST segment.

**Conclusions:**

EIANet demonstrates promising potential for accurately predicting EDCA using triage ECG images, offering an effective solution for early detection of high-risk cases in emergency settings. This approach may enhance the ability of health care professionals to make timely decisions, with the potential to improve patient outcomes by enabling earlier interventions for EDCA.

## Introduction

In-hospital cardiac arrest (IHCA) differs significantly from out-of-hospital cardiac arrest (OHCA), with the former often occurring in patients with significant comorbidities rather than as sudden cardiac events [1-3]. Despite its clinical significance, IHCA has received less research attention. Recent studies using data from the American Heart Association’s Get With the Guidelines-Resuscitation registry revealed an IHCA incidence of approximately 10 per 1000 bed-days (290,000 cases annually), with a survival rate of 15% to 20% at hospital discharge [4,5]. Emergency department (ED)–based IHCA (EDCA), accounting for 10% to 20% of all IHCA cases [6], is associated with higher mortality and less well-defined causes than inpatient cardiac arrest (IPCA) [7]. ED crowding, coupled with infrequent monitoring and unstable conditions, further increases EDCA risk [8].

Most patients experiencing IHCA show clinical deterioration hours before arrest, leading to the adoption of rule-based early warning scores to detect high-risk individuals [9-11]. With advancements in information technology, the use of machine learning for predicting IHCA has become increasingly prominent. Recent studies have demonstrated the potential of machine learning and statistical approaches in disease prediction, including heart disease analysis [12]. Although some deep learning models using structured data, such as vital signs [13-15], exist for predicting IPCA, they are not tailored for ED-specific conditions, highlighting the need for a dedicated ED-focused predictive model to address unique challenges in this setting. Using both triage and vital sign time-series data, we recently developed and validated a deep learning–based prediction tool for EDCA [16].

Electrocardiogram (ECG) is an old tool in clinical medicine but has re-emerged for the prediction of low left ventricular ejection fraction [17], arrhythmia [18], dyskalemia [19], or even longer-term mortality [20]. A recent randomized controlled trial using artificial intelligence–enabled ECG to identify hospitalized patients with a high risk of mortality found that the implementation of the artificial intelligence–enabled ECG alert was associated with a significant reduction in all-cause mortality within 90 days [21]. This finding is somewhat surprising, as a recent Cochrane review did not find strong evidence supporting the utility of early warning systems and rapid response systems for the prevention of patient deterioration in acute adult hospital wards [22]. To our knowledge, only 1 study has analyzed ECGs solely from inpatients to predict cardiac arrest within 24 hours of ECG acquisition [23]. This study analyzed signal-based ECGs, which require complex signal processing before analysis. In addition, XML-based protocols, commonly used by several ECG device manufacturers, store structured ECG data and enable integration with systems like General Electric’s MUSE server for advanced diagnostics. However, such reliance on proprietary software and infrastructure limits their applicability in the ED, which has diverse device environments. In contrast, image-based 12-lead ECG models leverage universally available PDF reports generated at ED triage. This approach eliminates the need for additional preprocessing or proprietary systems, providing a more accessible, flexible, and scalable solution that seamlessly integrates into real-world clinical workflows.

Accordingly, this study explored an innovative approach by using triage 12-lead ECG images as a more accessible alternative for predicting imminent cardiac arrest in the ED setting. We leveraged ECG reports, commonly available in PDF, and converted them into image-based data inputs to develop a deep learning–based predictive system for IHCA. We also used 2 independent ED data sets for development and validation of our model.

## Methods

### System Overview

**Figure 1:**
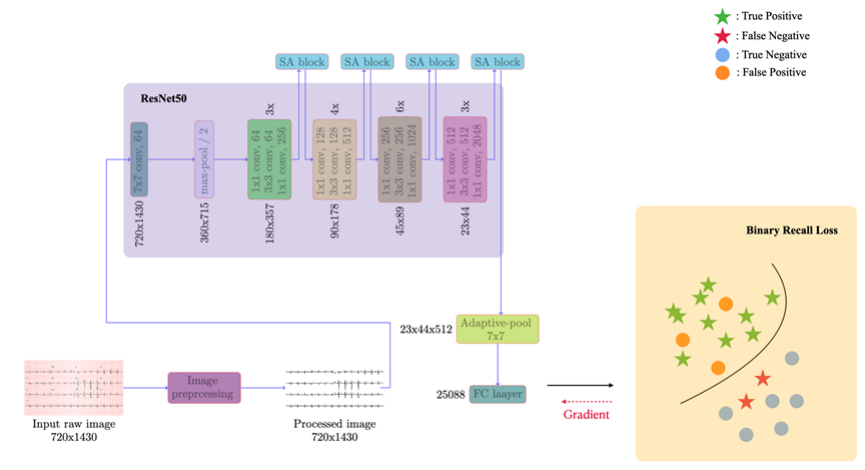
Overview of the proposed ECG-Image-Aware Network (EIANet) model. SA: spatial attention.

We present a novel cardiac arrest prediction model called the ECG-Image-Aware Network (EIANet) that was designed to predict IHCA with high accuracy. Our study adhered to the Guidelines for Developing and Reporting Machine Learning Predictive Models in Biomedical Research [24]. EIANet operated in 3 key stages, as outlined in Figure 1. In the first stage, ECG waveform images were extracted from triage ECG reports obtained from the National Taiwan University Hospital (NTUH). The NTUH is a tertiary academic medical center with approximately 2400 beds and 100,000 ED visits per year. We obtained 12-lead ECG images at ED triage for adult patients with EDCA (cases) from 2011 to 2019. We defined EDCA as patients who arrived in the ED with vital signs and later developed cardiac arrest in the ED. OHCAs with or without return of spontaneous circulation on ED arrival were excluded. We focused on treated EDCA, so patients with a do-not-resuscitate order were also excluded. The EDCA cases were from the same pool as in our previous studies on EDCA [16,25]. Control ECGs were randomly selected from adult ED patients without cardiac arrest during the same study period.

The ECG images were subjected to a thorough preprocessing step, where extraneous information, artifacts, and noise were removed, ensuring that the data fed into the model were clean and of high quality for more accurate analysis. In the second stage, the preprocessed ECG images were passed through a deep learning model, which was enhanced using a spatial attention module. This module allowed the model to focus on more relevant areas of the ECG image, identifying subtle patterns and features critical for predicting cardiac arrest. Finally, in the third stage, the model’s output was combined with the recall metric to calculate the binary recall loss (BRLoss). This custom loss function helps address slight class imbalance by prioritizing recall during training, leading to more balanced predictions, particularly in rare IHCA cases.

### ECG Data Set for IHCA Prediction

#### NTUH-ECG Data Set

The NTUH-ECG data set is a comprehensive collection of ECG data sourced from ED patients at NTUH, covering a significant period from 2011 to 2019. During this period, the data set was meticulously curated to include as many positive instances of EDCA as possible, representing critical cases that pose serious risks to patient safety. To balance the data set, random samples of ECGs from patients who did not experience EDCA were collected to serve as negative examples. Distinct from conventional ECG data sets that primarily focus on raw signal data, the NTUH-ECG data set features detailed ECG PDF reports containing 12-lead waveforms (I, II, III, aVR, aVL, aVF, and V1-V6) captured over 10-second intervals at ED triage. The primary objective of this data set is to predict the likelihood of a patient experiencing cardiac arrest during the ED stay, a task of immense clinical significance. The data set comprised a total of 571 cardiac arrest events (positive samples) and 826 noncardiac arrest events (negative samples), resulting in a positive sample ratio of 40.1%. For model training and evaluation, the data were randomly split into training and testing sets, with 80% allocated for training and 20% allocated for testing. This division yielded a test set containing 280 data points, which included 114 positive cases and 166 negative cases. The key characteristics of the NTUH-ECG data set are summarized in Table 1, providing valuable insights into its structure and composition for researchers and clinicians alike. The median time to cardiopulmonary resuscitation was approximately 5 to 7 hours for the cases and controls.

**Table 1:** Basic characteristics of the training and testing data sets for the National Taiwan University Hospital electrocardiogram (NTUH-ECG) data set.

Variable	NTUH-ECG training data set (n=1117)	NTUH-ECG testing data set (n=280)
Age (years), mean (SD)	65.9 (15.9)	63.7 (17.2)
Male, n (%)	608 (54.4)	141 (50.4)
Annotated positive, n (%)	457 (40.9)	114 (40.7)
Time to CPR^a^ (hours), median (IQR)	7.4 (2.5-23.5)	5.1 (1.9-17.9)
Annotated negative, n (%)	660 (59.1)	166 (59.3)

^a^CPR: cardiopulmonary resuscitation.

#### Far Eastern Memorial Hospital ECG Data Set

The Far Eastern Memorial Hospital (FEMH)-ECG data set consisted of ECG images collected from ED patients presenting to the FEMH from 2016 to 2023. The FEMH is a tertiary academic medical center with approximately 1200 beds and 130,000 ED visits per year. We obtained 12-lead ECG images at ED triage for adult patients with ED cardiac arrest (cases) from 2016 to 2023. Control ECGs were randomly selected from adult ED patients without cardiac arrest during the same study period. Each image was carefully annotated to indicate whether the patient experienced an EDCA during their ED stay. The data format closely mirrors that of the NTUH-ECG data set, with the images being directly extracted from ECG PDF reports. However, the distribution of positive samples in FEMH-ECG was significantly lower than in NTUH-ECG, at 378 positive cases and 713 negative cases, which equates to a positive sample proportion of approximately 34.6%. Table 2 shows the basic characteristics of the FEMH-ECG data set. The median time to cardiopulmonary resuscitation was approximately 2.5 hours.

**Table 2:** Basic characteristics of the positive and negative samples for the Far Eastern Memorial Hospital electrocardiogram (FEMH-ECG) data set.

Variable	FEMH-ECG positive samples (n=378)	FEMH-ECG negative samples (n=713)
Age (years), mean (SD)	68.3 (14.9)	64.4 (17.9)
Male, n (%)	252 (66.7)	389 (54.6)
Time to CPR^a^ (hours), median (IQR)	2.5 (0.7-8.9)	—^b^

^a^CPR: cardiopulmonary resuscitation.

^b^Not applicable.

### Ethical Considerations

The NTUH study was approved by the NTUH Institutional Review Board (reference number: 202304129RINC), which waived the requirement for patient informed consent. The FEMH study was approved by the FEMH Institutional Review Board (reference number: 112193-F), which also waived the requirement for patient informed consent.

### Preprocessing

Although deep learning models can automatically extract relevant features from input data, their performance can be significantly improved when noisy or unclean data are addressed through careful preprocessing. In our approach, we began by converting the RGB ECG image into a binary format using a thresholding technique. Specifically, we first used Gaussian blur and thresholding to remove unnecessary elements such as thin grid lines. By applying a threshold, pixels exceeding the threshold value were turned black (0), while those below the threshold were converted to white (255). This step ensured that distracting elements were removed, enhancing the clarity of the ECG waveform for further analysis.

Next, we used a connected component algorithm to identify and remove specific unwanted components such as lead labels and other extraneous noise in the image. These elements, if left unaddressed, can interfere with the accuracy of the model’s predictions. To further refine the image, we applied a morphological opening operation, which consists of erosion followed by dilation, using a 3 × 3 structuring element. This process smoothed the ECG lines and reduced minor noise, ensuring that the primary features of the ECG waveform were preserved while removing small, irrelevant artifacts. This enhanced the overall quality of the image, making it more suitable for deep learning analysis.

To complete the preprocessing, we applied a Gaussian blur to the image, which helped minimize any remaining noise that may have been left after previous steps. The ECG image was then transformed to gray scale, and we used histogram equalization to improve the contrast between the ECG waveform and any residual background noise. This step ensured that the critical features of the waveform were more distinct, allowing the deep learning model to focus on the most relevant data points. In the final stage of preprocessing, the image was reconverted to binary format, accentuating the essential features necessary for more accurate and effective analysis by the model, ultimately improving its ability to predict IHCA.

### Spatial Attention

In our model, we used the ResNet50 [26] architecture as the foundational framework due to its robust performance in handling complex image data. ResNet50’s use of residual connections enhances the learning capacity of deep networks by facilitating the flow of information and mitigating issues such as vanishing gradients. We integrated an attention mechanism into the network. This integration ensured that the model can better identify subtle, critical signals in the ECG data, ultimately enhancing its predictive performance and improving patient outcomes.

**Figure 2:**
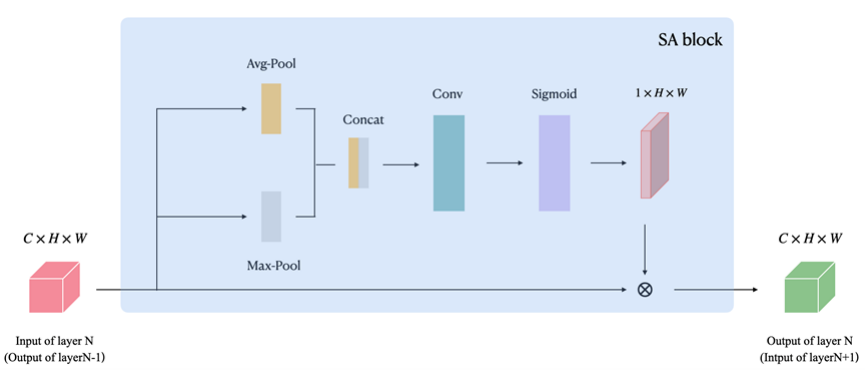
We inserted a spatial attention (SA) block after each layer and before the next layer.

Following the concept of the Convolutional Block Attention Module [27], we strategically positioned spatial attention blocks after each of the first 4 layers, as Figure 2. This design choice was motivated by 2 primary considerations. First, the nature of ECG waveforms, which display both fine and coarse-grained patterns, necessitates a multiscale approach to feature extraction. By placing spatial attention blocks at the front, middle, and end of the network, we enhanced the model’s ability to capture and optimize features across varying scales. This is crucial for accurately interpreting the diverse characteristics present in ECG signals, which can vary significantly in amplitude and duration due to different physiological conditions. Second, we intentionally limited the number of attention blocks to mitigate the risk of overfitting during the training process. By judiciously inserting these blocks after each layer, we balanced the complexity of the model with its generalization capabilities.

The incorporation of the spatial attention mechanism allowed the model to learn more representative features, leading to improved performance. Visualization results further demonstrated the model’s ability to concentrate on more relevant patterns. Detailed findings are discussed in the following section.

### Binary Recall Loss

Our training data set exhibited a slight imbalance of positive and negative samples, which posed a challenge for accurately predicting IHCA. Given the serious threat that IHCA presents to patient safety, maximizing the model’s recall is of paramount importance, as it measures the system’s ability to correctly identify true positive cases. A high recall ensures that most actual cardiac arrest occurrences are detected, allowing health care professionals to respond swiftly and effectively. However, this focus on recall can inadvertently lead to an increase in false positives, overwhelming the health care system with unnecessary alarms and interventions. Such a scenario can strain resources and divert attention from genuine emergencies, potentially leading to alarm fatigue among medical staff. Therefore, it is equally critical to maintain a reasonable level of precision alongside recall. Striking a balance between these 2 metrics is essential to ensure that we effectively identify critical cases of IHCA while also minimizing the burden on health care providers, ultimately safeguarding patient safety and enhancing the efficiency of emergency care.

To address this, rather than using the traditional binary cross-entropy loss, we used BRLoss by drawing on the concept of performance-based loss from [28]. BRLoss used recall-related weights for each class, as shown in Equation 1:



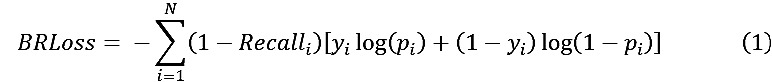



where N is the number of samples in a batch, *y_i_* is the ground truth label of i-th data in the batch, *y_i_* ∈ [0, 1], *p_i_* is the predicted probability, 0 ≤ *p_i_* ≤ 1 and *Recall_c_* means recall of class c, *Recall_c_* [0, 1], c is 0 or 1.

Unlike many other statistics-based weighted loss [29-31] functions that may cause unnecessary false positives, sacrificing precision to gain recall, BRLoss effectively balanced recall and precision. We used the same analytical method as [26] to perform a partial derivative of the loss function. Assuming the *i* – *th* data sample in the batch has a final output *z_i_* before applying the sigmoid function, the gradient for BRLoss can be calculated as shown in Equation 2:



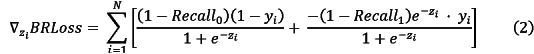



In the case where the ground truth is 1, an increase in false negatives leads to a larger negative gradient in the term related to *Recall*_1_in Equation 2. This drives *z* to increase during gradient descent, reducing false negatives and improving *Recall*_1_. Similarly, when the ground truth is 0, more false positives result in a larger positive gradient in the term related to *Recall*_0_ (specificity), causing *z* to decrease and reduce false positives. BRLoss thus balanced recall and precision, leading to more reliable model performance.

## Results

We performed experiments using the NTUH-ECG and FEMH-ECG data sets. Given the increasing significance of explainable artificial intelligence, particularly in the medical domain, interpretability had become essential [32-34]. Therefore, we present various qualitative results and conducted ablation studies to clarify model behavior under different conditions.

### Implementation Details

We implemented EIANet using the Python and Pytorch [35] framework. The EIANet training was accelerated using an RTX 3090 GPU. The AdamW [36] optimizer was used to train the model over 200 epochs, with a learning rate initialized at 2 × 10^-5^.

### Evaluation Metrics for IHCA Prediction

In our binary classification task aimed at predicting IHCA using ECG images, we used a comprehensive evaluation strategy to assess model performance. This involved using several metrics derived from the confusion matrix, such as accuracy, precision, recall (sensitivity), and specificity. These metrics provided a detailed breakdown of how well the model distinguished between positive (IHCA) and negative (non-IHCA) cases, helping us understand not only the overall accuracy but also how well the model balanced false positives and false negatives. We also calculated the area under the receiver operating characteristic curve (AUROC), which measured the trade-off between true positive rates and false positive rates across different decision thresholds. A higher AUROC value indicates that the model is more capable of distinguishing between the 2 classes, even in the presence of class imbalance. Moreover, we incorporated the area under the precision-recall curve (AUPRC) to evaluate performance, particularly in handling imbalanced data sets like ours, where the positive class is underrepresented. The AUPRC focuses on precision (positive predictive value) and recall, offering a more sensitive measure of the model’s performance when identifying IHCA cases without being skewed by the larger number of negative cases. Together, these metrics provided a robust, multifaceted assessment of our model’s effectiveness at predicting IHCA, ensuring that it performs well across a range of critical evaluation criteria.

### Experimental Results With ECG Data Sets

#### External Comparison

Table 3 presents a comprehensive comparison between our study and the work of Kwon et al [23], which used raw ECG signals to predict IPCA. In addition to differences in patient populations (ED vs inpatients), a key distinction between the 2 studies lies in the format of the input data: Although Kwon et al [23] used raw ECG signals, our approach was built around the use of ECG images. This shift from signal-based input to image-based input enables our model to leverage advanced image processing techniques, such as spatial attention, to enhance feature extraction. In contrast, raw signals can be directly analyzed using the temporal relationships between successive values.

**Table 3:** Comparison of our study with Kwon et al [23], highlighting differences in input data formats (electrocardiogram images vs raw signals) and data set balance.

Method	Positive rate	Accuracy	Recall	Precision	Specificity	AUROC^a^
Kwon et al [23]	0.0093	0.918	0.778	0.076	0.920	0.948
Ours	0.401	0.843	0.800	0.813	0.884	0.896

^a^AUROC: area under the receiver operating characteristic curve.

Another significant difference is the distribution of positive samples between the 2 data sets. The work by Kwon et al [23] involved a highly imbalanced data set with a larger proportion of negative samples, which is reflected in their higher specificity. In contrast, our data set was more balanced, with a more equitable distribution of positive and negative samples, which had a direct influence on the performance metrics. This difference in the proportion of positive samples played a crucial role in shaping outcomes like precision, accuracy, and AUROC, making direct comparisons between the 2 models less straightforward.

In summary, our approach, with spatial attention, BRLoss, and more balanced sample distribution, can enhance the accurate detection of positive cases, leading to a slightly higher recall. The model by Kwon et al [23], having a predominantly negative sample set, excels in specificity but may sacrifice sensitivity to positive cases. The distinct methodologies and differing data set characteristics highlight the complementary strengths of the 2 approaches, offering insights into how input formats and sample distributions can impact the prediction performance of cardiac arrest models.

#### Comparison With the Benchmark

To the best of our knowledge, the NTUH-ECG data set stands as the only data set we can get to date that uses image-based ECG data specifically for the prediction of EDCA. Our proposed model, EIANet, represents a pioneering approach in this domain, being the first to tackle this predictive task using ECG images as input. We used the well-established vision model ResNet50 as a baseline for comparative analysis, leveraging its widespread recognition in image-based tasks as a point of reference.

Table 4 shows a side-by-side comparison of the performance of EIANet and ResNet50. Compared with solely using the ResNet50 module, EIANet incorporates spatial attention, enabling the model to focus more on spatially crucial features. With stronger feature representations, combined with BRLoss, the model’s ability to differentiate between positive and negative samples is further enhanced. IEIANet significantly outperforms ResNet50 across multiple evaluation metrics, underscoring the efficacy of our tailored approach. We attribute this marked improvement to 2 key innovations in our model design. First, the incorporation of the spatial attention module substantially enhanced the model’s feature extraction capabilities. By using spatial attention, EIANet can dynamically focus on the most relevant regions within the ECG images, allowing it to capture critical visual patterns that are often indicative of impending cardiac events. This focused attention provides the model with a more nuanced understanding of the subtle yet vital features present in ECG images. In addition to spatial attention, BRLoss helps to mitigate the risk of too many false positives and false negatives, leading to a slight improvement in recall without affecting precision too much.

**Table 4:** Comparison of our ECG-Image-Aware Network (EIANet) module with the baseline (ResNet50) module.

Method	Accuracy	Recall	Precision	*F*_1_-score	AUROC^a^	AUPRC^b^
Baseline (ResNet50)	0.786	0.763	0.725	0.744	0.846	0.789
Ours (EIANet)	0.843	0.800	0.813	0.805	0.896	0.842

^a^AUROC: area under the receiver operating characteristic curve.

^b^AUPRC: area under the precision-recall curve.

### Qualitative Results

**Figure 3:**
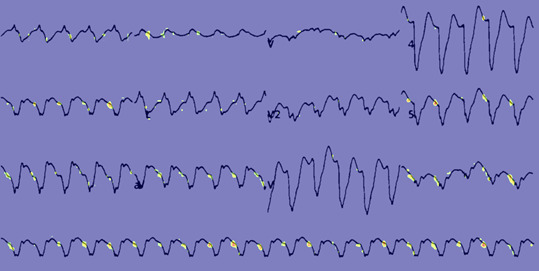
Feature map of a National Taiwan University Hospital electrocardiogram (NTUH-ECG) sample with a spatial attention block.

**Figure 4:**
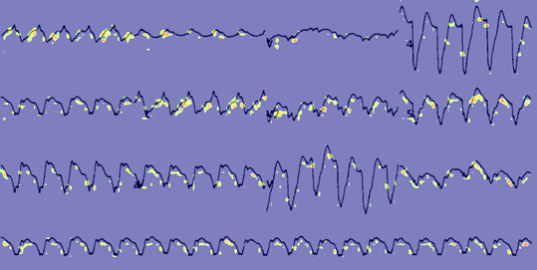
Feature map of a National Taiwan University Hospital electrocardiogram (NTUH-ECG) sample without a spatial attention block.

Figure 3 and Figure 4 provide feature map [37] visualizations of positive samples from the NTUH-ECG data set. The visualization shows the Grad-CAM output, highlighting regions with Grad-CAM values greater than 0.4. Figure 3 shows the output of a model incorporating spatial attention, while Figure 4 presents the results of a model devoid of spatial attention. Alterations in the ST segment serve as pivotal indicators of myocardial ischemia. Notwithstanding the presence of a bundle branch block in this ECG, EIANet, as depicted in Figure 3, could still accurately pinpoint the ST segment. This underscores EIANet’s capacity to discern patterns associated with EDCA within ECG images. Furthermore, tachycardia is a prevalent ECG manifestation preceding EDCA. The regular bright spots in the figures suggest that the algorithm is adept at counting the heart rate. In contrast, although the model without spatial attention, as illustrated in Figure 4, also counted the heart rate, its focus on the ST segment was less precise and more dispersed.

**Figure 5:**
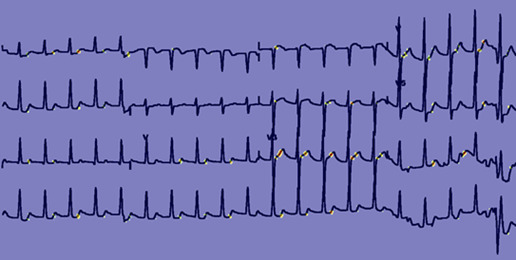
Feature map of a Far Eastern Memorial Hospital electrocardiogram (FEMH-ECG) sample with a spatial attention block.

**Figure 6:**
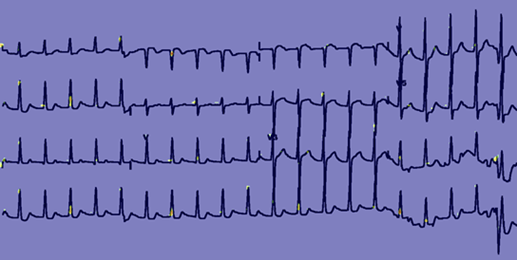
Feature map of a Far Eastern Memorial Hospital electrocardiogram (FEMH-ECG) sample without a spatial attention block.

Figure 5 and Figure 6 present visualizations from the FEMH-ECG data set, which further illustrate the impact of spatial attention by focusing on heart rate–related features. In Figure 5 (with spatial attention), the model effectively identifies regular bright spots corresponding to heartbeats, indicating its ability to count the heart rate accurately. Conversely, Figure 6 (without spatial attention) shows a less distinct attention pattern, reducing interpretability. 

### Ablation Study

The first ablation study focused on evaluating the impact of different types of input data on model performance. As detailed in Table 5, using raw ECG images directly extracted from PDF reports for training demonstrated a certain level of predictive capability, indicating that even unprocessed images contain valuable information that the model can learn from. However, the study revealed that, when these raw images undergo a series of image preprocessing steps to remove irrelevant or distracting elements, the model’s performance saw marked improvement because the model could focus on crucial visual patterns and structures essential for predicting EDCA. This performance boost underscores the importance of preprocessing for enhancing the quality of the input data.

**Table 5:** Comparison of raw images and processed images on the National Taiwan University Hospital electrocardiogram (NTUH-ECG) data set.

Input	Accuracy	Recall	Precision	*F*_1_-score	AUROC^a^	AUPRC^b^
Raw image	0.782	0.737	0.718	0.727	0.832	0.785
Processed image	0.843	0.800	0.813	0.805	0.896	0.842

^a^AUROC: area under the receiver operating characteristic curve.

^b^AUPRC: area under the precision-recall curve.

By applying preprocessing techniques, such as noise reduction, removal of grid lines, and extraction of key features, the model was better able to focus on the most relevant aspects of the ECG images, allowing it to learn more effectively. Preprocessing not only reduced the amount of noise and irrelevant information present in the images but also helped to clarify the visual patterns and structures that are crucial for predicting EDCA. This led to more accurate predictions and overall model performance.

Table 6 presents an ablation study evaluating the influence of spatial attention and BRLoss on the model’s performance and demonstrates the impact of incorporating the spatial attention mechanism and BRLoss on model performance. We trained our model on the NTUH-ECG training set and validated it using both the NTUH-ECG testing set and the FEMH-ECG data set. As expected, due to differences in patient populations, there was a slight decrease in model performance in the FEMH-ECG data set. The results indicate that the spatial attention module and BRLoss elevated the model’s performance. The spatial attention mechanism empowered the model to extract more effective ECG features, such as the ST segment and heart rate patterns, thereby enhancing feature representation and overall model accuracy. This mechanism ensured that the model concentrated on critical segments of the ECG image, leading to improved interpretability and reliability. Moreover, BRLoss ameliorated the model’s recall without compromising precision to a significant degree. BRLoss focuses on reducing false predictions and improving *F*_1_-score and AUROC. However, its effectiveness also depends on the model’s ability to sufficiently represent the data. When combined with spatial attention, the performance of the model can be further enhanced by leveraging the strengths of both modules. Although BRLoss prioritizes recall, decision thresholds can be adjusted in clinical applications to achieve a suitable balance between recall and precision, addressing specific requirements in emergency scenarios.

**Table 6:** Ablation study results of modules in ECG-Image-Aware Network (EIANet).

Modules		Accuracy	Recall	Precision	*F*_1_-score	AUROC^a^	AUPRC^b^
**NTUH-ECG** ^c^ **data set**							
	None	0.742	0.728	0.670	0.700	0.841	0.792
	SA^d^ only	0.825	0.789	0.783	0.786	0.874	0.825
	BRLoss^e^ only	0.796	0.781	0.736	0.757	0.869	0.816
	SA + BRLoss	0.843	0.800	0.813	0.805	0.896	0.842
**FEMH-ECG** ^f^ **data set**							
	None	0.707	0.626	0.571	0.597	0.781	0.621
	SA only	0.734	0.645	0.610	0.627	0.814	0.697
	BRLoss only	0.726	0.613	0.602	0.607	0.797	0.676
	SA + BRLoss	0.743	0.688	0.616	0.650	0.803	0.678

^a^AUROC: area under the receiver operating characteristic curve.

^b^AUPRC: area under the precision-recall curve.

^c^NTUH-ECG: National Taiwan University Hospital electrocardiogram.

^d^SA: spatial attention.

^e^BRLoss: binary recall loss.

^f^FEMH-ECG: Far Eastern Memorial Hospital electrocardiogram.

### Ablation Study With Varying Class Imbalances

To evaluate the contributions of the spatial attention module and BRLoss under varying class imbalances, we performed random resampling of the NTUH-ECG and FEMH-ECG data sets to adjust the proportion of positive samples to 0.1 and 0.2. Each resampling experiment was repeated 30 times, and the results were averaged to ensure robustness. The ablation study results under positive sample ratios of 0.1 and 0.2 are presented in Table 7. These results clearly demonstrate the contributions of spatial attention and BRLoss to the model’s performance. In addition, for metrics that are not dependent on the prevalence of positive cases (eg, AUROC), the model performance remained.

**Table 7:** Ablation study results of modules in ECG-Image-Aware Network (EIANet).

Modules		Accuracy	Recall	Precision	*F*_1_-score	AUROC^a^	AUPRC^b^
**NTUH-ECG** ^c^ **data set:** **positive ratio of 0.1**							
	None	0.749	0.714	0.238	0.357	0.837	0.436
	SA^d^ only	0.843	0.783	0.359	0.492	0.871	0.491
	BRLoss^e^ only	0.805	0.780	0.304	0.438	0.872	0.495
	SA + BRLoss	0.865	0.788	0.402	0.532	0.894	0.493
**FEMH-ECG** ^f^ **data set:** **positive ratio of 0.1**							
	None	0.737	0.621	0.215	0.319	0.787	0.265
	SA only	0.767	0.637	0.243	0.351	0.811	0.368
	BRLoss only	0.769	0.605	0.237	0.340	0.794	0.335
	SA + BRLoss	0.764	0.685	0.250	0.366	0.803	0.339
**NTUH-ECG data set:** **positive ratio of 0.2**							
	None	0.747	0.728	0.421	0.533	0.842	0.612
	SA only	0.840	0.800	0.567	0.664	0.875	0.660
	BRLoss only	0.802	0.780	0.499	0.609	0.869	0.646
	SA + BRLoss	0.858	0.798	0.608	0.690	0.895	0.674
**FEMH-ECG data set:** **positive ratio of 0.2**							
	None	0.726	0.629	0.386	0.319	0.780	0.444
	SA only	0.754	0.647	0.425	0.351	0.814	0.644
	BRLoss only	0.750	0.608	0.414	0.340	0.797	0.515
	SA + BRLoss	0.755	0.686	0.429	0.366	0.803	0.515

^a^AUROC: area under the receiver operating characteristic curve.

^b^AUPRC: area under the precision-recall curve.

^c^NTUH-ECG: National Taiwan University Hospital electrocardiogram.

^d^SA: spatial attention.

^e^BRLoss: binary recall loss.

^f^FEMH-ECG: Far Eastern Memorial Hospital electrocardiogram.

## Discussion

### Principal Findings

To our knowledge, this is the first study to develop and validate a deep learning algorithm (EIANet) specifically for predicting EDCA using ECG image data. The findings demonstrate that deep learning, a powerful artificial intelligence tool, can detect subtle ECG changes hours before cardiac arrest in the ED. Deployment of this tool at ED triage has the potential to gain lead time to identify high-risk patients for timely interventions and reduce EDCAs.

### Comparison With Prior Work

Traditionally, ECG prediction of cardiac arrest has mainly focused on OHCA (ie, using ECG to predict sudden cardiac death at the population level) [38]. For example, an electrical risk score that comprises 6 features (heart rate, prolonged corrected QT interval, Tpeak-Tend interval, QRS-T angle, left ventricular hypertrophy, and delayed QRS transition zone) can accurately predict sudden cardiac death within a community-based cohort [39]. A refined deep learning–based ECG model can further improve its predictive ability using the same 6 features [40]. Notably, tachycardia has been included as one of the features in the electrical risk score and was also documented in our feature map analysis and in a previous IPCA ECG study [23], suggesting the importance of this feature in predicting OHCA, IPCA, and EDCA.

One of our previous studies on EDCA focused on the development and validation of a simple 8-item, rule-based prediction tool using structural data at ED triage [25]. Another study of ours further advanced EDCA prediction by using deep learning with static triage data and dynamic vital signs measured during the ED stay [16]. None of these studies used ECG as an input for deep learning, as we did in this study. Given the promising results of using triage ECG to predict EDCA, the next logical step would be to combine ECG and structural data as a multimodal model for EDCA prediction.

### Strengths

The EIANet has several technical innovations. Before model training, we used image processing techniques to eliminate manual labels and noise from the ECG images. To enhance model performance, we integrated an attention mechanism and BRLoss. These components had a positive impact on EIANet’s predictive capabilities. Our research used 2 ECG image data sets: NTUH-ECG and FEMH-ECG. The NTUH-ECG data set, with a positive sample ratio of 40%, yielded impressive results, with EIANet achieving an *F*_1_-score of 0.805 and AUPRC of 0.842. The FEMH-ECG data set, characterized by a lower positive sample ratio of 34.6%, still demonstrated strong performance, with EIANet attaining an *F*_1_-score of 0.650 and AUPRC of 0.678. To validate the effectiveness of the proposed modules, we conducted ablation studies, further confirming their importance. Additionally, Grad-CAM visualization was used to provide interpretability for EIANet’s predictions using ECG images.

In the past, ECG prediction models were typically designed for diseases with relatively obvious features, such as myocardial infarction or hyperkalemia [41,42]. However, precardiac arrest ECGs lack absolutely distinct characteristics, making EDCA significantly more challenging to predict. Additionally, most existing image-based ECG models have remained in the proof-of-concept stage, often relying on general purpose framework models like ResNet without substantial customization for this specific task. These approaches, although promising, have struggled with critical limitations, such as high false-negative rates and limited interpretability. To address these challenges, we enhanced ResNet50 by incorporating a spatial attention mechanism, enabling the model to focus on subtle but clinically significant ECG patterns. Furthermore, we introduced a BRLoss function to prioritize recall and reduce false negatives, a critical improvement for predicting rare but life-threatening events like EDCA. These innovations aimed to overcome the inherent limitations of prior approaches and establish a practical, interpretable, and effective prediction framework.

### Limitations and Future Directions

Although our results demonstrate a certain level of predictive capability on both the NTUH-ECG and FEMH-ECG data sets, the performance on the FEMH-ECG data set declined. The observed drop in recall when evaluating the EIANet model on the FEMH-ECG data set can be attributed to multiple factors. First, patient population differences between the data sets likely played a significant role. Variations in patient populations could influence the manifestations of ECGs, leading to differing model performance. Second, differences in instrumentation between the 2 data sets may have introduced some biases. These discrepancies, combined with slight differences in image sizes formats and preprocessing process, might have impacted the model’s ability to generalize effectively. Addressing these challenges in future work by incorporating more diverse data sets could enhance the model’s robustness and applicability across different clinical settings. In the future, we hope to use EIANet as an encoder for ECG data, integrating it with other modalities of patient information to establish a more comprehensive multimodal prediction method for EDCA.

This study has some potential limitations. First, the model’s performance was dependent on the quality and variety of the training data. Although we included ECGs from 2 independent medical centers, further external validation studies are needed to evaluate the model’s robustness in different patient populations. Second, this was a retrospective study. Even with external validation, as in our retrospective study, models could have poor performance in the prospective setting, such as the Rothman Index by PeraHealth [43,44] and the Epic Sepsis Model [45,46]. Future implementation of the EIANet model in the ED would be warranted to test its real-world effectiveness.

### Conclusions

In conclusion, we presented a comprehensive study aimed at understanding the relationship between ECG images and EDCA. To the best of our knowledge, this is the first in the literature that uses readily available 12-lead ECG images at ED triage to predict imminent ED cardiac arrest. Using 2 independent ED data sets, we developed and externally validated the novel deep learning EIANet model that can be used to identify high-risk patients and potentially reduce devastating ED cardiac arrest events. Future implementation of the EIANet in real time is warranted to test whether this tool could flag high-risk patients at ED triage for timely interventions, thereby improving patient outcomes.

## Data Availability

The artificial intelligence algorithm cannot be made publicly available because it is proprietary intellectual property. The computer code and data for this study are available from the corresponding authors upon reasonable request for research studies.

## Abbreviations

AUPRC: area under the precision-recall curve
AUROC: area under the receiver operating characteristic curve
BRLoss: binary recall loss
ECG: electrocardiogram
ED: emergency department
EDCA: emergency department–based in-hospital cardiac arrest
EIANet: ECG-Image-Aware Network
FEMH: Far Eastern Memorial Hospital
IHCA: in-hospital cardiac arrest
IPCA: inpatient cardiac arrest
NTUH: National Taiwan University Hospital
OHCA: out-of-hospital cardiac arrest
